# Differential Effects of Donepezil and Tacrine on Recall-Phase Exploratory Behavior in Healthy Male Wistar Rats: A Two-Trial Y-Maze Study

**DOI:** 10.3390/biom16081102

**Published:** 2026-07-28

**Authors:** Adrian-Florentin Dragomir, Smaranda Stoleru, Aurelia Cristiana Barbu, Aurelian Zugravu, Maria Carina Dumitrescu, Miruna Valeria Moraru, Laurentiu-Nicolae Ciuca, Silvia Fratea, Claudia Elena Dobre, Clara Maria Stoleru, Oana Andreia Coman, Ion Fulga

**Affiliations:** 1 Faculty of Medicine, “Carol Davila” University of Medicine and Pharmacy, 050474 Bucharest, Romania; adrian-florentin.dragomir@drd.umfcd.ro (A.-F.D.); smaranda.stoleru@umfcd.ro (S.S.); maria-carina.dumitrescu@rez.umfcd.ro (M.C.D.); miruna-valeria.moraru@drd.umfcd.ro (M.V.M.); nicolae.ciuca@umfcd.ro (L.-N.C.); oana.coman@umfcd.ro (O.A.C.); ion.fulga@umfcd.ro (I.F.); 2 Service Anesthesie-Reanimation, Groupe Hospitalier de la Pitié-Salpêtrière, CEDEX 13, 75651 Paris, France; silvia.fratea@aphp.fr; 3 Faculty of Midwifery and Nursing, “Carol Davila” University of Medicine and Pharmacy, 050474 Bucharest, Romania; claudia.dobre@umfcd.ro; 4 Faculty of Medical Engineering, National University of Science and Technology Politehnica Bucharest, 060042 Bucharest, Romania; clara_maria.stoleru@stud.fim.upb.ro

**Keywords:** cholinesterase inhibitors, donepezil, tacrine, Y-maze, exploratory allocation, spatial exploration, male Wistar rats

## Abstract

Cholinesterase inhibitors with distinct pharmacological profiles may influence exploratory behavior, yet their effects on recall-related exploration in physiologically intact animals remain insufficiently characterized. The present study aimed to directly compare the effects of donepezil, a relatively selective acetylcholinesterase inhibitor, and tacrine, a cholinesterase inhibitor with broader enzymatic and pharmacological activity, on exploratory allocation during memory recall in healthy rats. Male Wistar rats were assigned to five groups (*n* = 8/group): control, donepezil 1 mg/kg, donepezil 3 mg/kg, tacrine 3 mg/kg, and tacrine 5 mg/kg. Treatments were administered intraperitoneally 50 min before the acquisition trial, while recall performance was evaluated 24 h later without additional treatment using a two-trial Y-maze paradigm. Time spent and entries into the familiar and novel arms, novel-to-familiar ratios, and mean time per entry were analyzed. Control animals showed no marked preference for the novel arm, whereas donepezil produced only limited, non-significant behavioral changes. Tacrine 5 mg/kg significantly increased the U/K time ratio compared with controls (*p* = 0.0006) and the U/K entry ratio (*p* = 0.001), primarily through reduced time spent in the familiar arm (*p* = 0.0004) and fewer familiar-arm entries (*p* = 0.025). Absolute time spent in, and entries into, the novel arm did not differ significantly among groups (*p* = 0.689 and *p* = 0.883, respectively). Overall, these findings suggest that tacrine, but not donepezil, modified recall-phase exploratory preference in healthy rats, predominantly by reducing persistence in the familiar arm.

## 1. Introduction

Cholinergic neurotransmission plays an essential role in learning, memory, and exploratory behavior, particularly in hippocampal and cortical circuits involved in spatial processing and novelty-related responses [1,2]. Disruption of cholinergic neurotransmission is a well-recognized contributor to cognitive decline in neurodegenerative disorders, most notably Alzheimer’s disease, in which early degeneration of basal forebrain cholinergic neurons and reductions in choline acetyltransferase activity have been consistently reported [1,2,3,4].

Beyond its general modulatory function, cholinergic signaling plays a critical role in the processing of novel information [5,6]. Experimental studies using in vivo microdialysis have shown that cortical and hippocampal acetylcholine (ACh) release increases during exposure to novel stimuli and decreases as stimuli become familiar, indicating a link between cholinergic activity and stimulus novelty [4,5,7]. At the receptor level, both nicotinic and muscarinic acetylcholine receptors contribute to learning and memory processes. Pharmacological evidence indicates that muscarinic receptor blockade disrupts memory acquisition more consistently than memory retrieval, particularly when novel stimuli are involved, underscoring the importance of cholinergic signaling during the early stages of memory formation and encoding [5,6,7,8,9].

Acetylcholinesterase (AChE) inhibitors remain widely used for the symptomatic treatment of Alzheimer’s disease [10]. By limiting acetylcholine hydrolysis, these agents increase synaptic acetylcholine availability and enhance cholinergic neurotransmission, thereby partially compensating for cholinergic deficits [10,11]. Donepezil and tacrine are among the best-known representatives of this class and have demonstrated clinical efficacy in improving cognitive symptoms. Beyond their symptomatic role, some cholinesterase inhibitors have also been reported to exert broader neurobiological effects, including modulation of neuronal survival and synaptic function [1,10,11,12,13].

In the mammalian brain, two major enzymes—acetylcholinesterase (AChE) and butyrylcholinesterase (BuChE)—participate in ACh hydrolysis [4,14,15,16]. AChE is the principal enzyme responsible for synaptic ACh breakdown under physiological conditions, whereas BuChE exhibits a distinct cellular distribution and can also contribute to ACh metabolism [17,18]. Although BuChE accounts for a smaller proportion of total cholinesterase activity in the healthy brain, its relative activity increases in advanced Alzheimer’s disease, where AChE activity declines and BuChE levels rise. These alterations in cholinesterase balance suggest that pharmacological agents with different selectivity profiles toward AChE and BuChE may differentially modulate cholinergic transmission [4,13,17,18,19,20]. These enzymatic differences provide a pharmacological framework for comparing cholinesterase inhibitors with distinct selectivity profiles.

Despite belonging to the same pharmacological class, donepezil and tacrine differ in their enzymatic selectivity. Donepezil acts primarily as a selective acetylcholinesterase inhibitor, whereas tacrine inhibits both acetylcholinesterase and butyrylcholinesterase. Although tacrine has limited therapeutic relevance because of its known hepatotoxicity and tolerability concerns during repeated clinical use, it remains informative as a pharmacological comparator in acute experimental studies. Accordingly, the present work was designed to compare short-term behavioral effects following single-dose pre-acquisition administration rather than to assess comparative clinical utility or repeated-dose systemic toxicity [10,11,12].

Most experimental investigations of cholinesterase inhibitors have been conducted in models of cholinergic dysfunction or neurodegeneration, in which their behavioral effects are interpreted primarily in relation to the attenuation of an established deficit [20,21,22,23]. Although such models are highly relevant for therapeutic research, the behavioral consequences of a pharmacological intervention may also be influenced by the underlying pathology, the severity of the induced impairment, and the specific experimental protocol. In addition, direct comparisons across independent studies are complicated by differences in animal models, doses, routes of administration, treatment schedules, retention intervals, and behavioral endpoints.

In physiologically intact animals, cholinergic enhancement is not intended to restore an established deficit. Rather, it may reveal compound-specific patterns of exploratory behavior under baseline conditions. A direct comparison of donepezil and tacrine within the same experimental framework may therefore help distinguish drug-related behavioral differences from variation associated with different experimental protocols. Such baseline characterization is complementary to findings obtained in models of cholinergic impairment. In the two-trial Y-maze paradigm, rodents are initially exposed to a restricted spatial configuration and are subsequently tested with access to all arms, allowing assessment of exploratory allocation between familiar and newly accessible spatial alternatives [24]. This pattern of exploration may reflect spatial recognition-like behavior, but it is also influenced by locomotor activity, anxiety-related responses, exploratory drive, persistence in previously visited areas, and decision allocation between available arms [24,25]. Accordingly, changes in arm preference should be interpreted cautiously and not exclusively as direct evidence of memory enhancement.

Thus, the specific contribution of the present study lies in the standardized direct comparison of donepezil and tacrine under identical experimental conditions, with simultaneous analysis of absolute arm-specific measures, ratio-based indices, and differential exploratory parameters. Based on this framework, the present study aimed to directly compare the effects of donepezil and tacrine on recall-session exploratory allocation in healthy rats using a two-trial Y-maze paradigm. Arm-specific measures of time spent and entries, together with relative indices of exploratory allocation, were used to characterize the behavioral profile associated with each treatment.

We hypothesized that donepezil and tacrine would produce distinct exploratory patterns during the 24 h session, consistent with their different pharmacological characteristics. The study was designed to characterize and compare the exploratory behavioral profiles associated with donepezil and tacrine under identical experimental conditions in healthy rats, thereby providing a standardized framework for evaluating their differential effects in an intact cholinergic system.

## 2. Materials and Methods

The experimental protocol received ethical approval from the Ethics Committee of the “Carol Davila” University of Medicine and Pharmacy, Bucharest, Romania (approval no. 13619/24.05.2024), and authorization from the National Sanitary Veterinary and Food Safety Authority (approval no. 9/05.07.2024). All procedures involving animals were performed in accordance with Romanian legislation, Directive 2010/63/EU on the protection of animals used for scientific purposes, and the principles of Replacement, Reduction, and Refinement. The study was conducted in accordance with the Animal Research: Reporting of In Vivo Experiments (ARRIVE) guidelines.

### 2.1. Animals

Male Wistar rats were supplied by the animal breeding facility of the “Carol Davila” University of Medicine and Pharmacy, Bucharest. At arrival, the animals were 6–7 weeks old, and at the time of behavioral testing they weighed 200–300 g. Before the start of the experiments, rats were acclimatized to the laboratory environment for at least 7 days. They were housed individually under standard conditions, with ad libitum access to food and water, natural ventilation, relative humidity maintained at 40–60%, and a natural light cycle consistent with institutional animal housing conditions. Animals were housed individually in order to minimize potential variability related to social interaction during exploratory testing, while environmental conditions were kept stable throughout the study.

Experimental procedures were carried out in the Department of Pharmacology between 10:00 and 17:00. To avoid repeated exposure to experimental conditions, each animal was included in only one experimental protocol. All animals were behaviorally naïve before the start of the study.

### 2.2. Experimental Design

Five experimental groups were included in the study (*n* = 8 per group), as follows:

(1) C = control group: 0.1 mL/100 g (i.p.), 0.9% saline.
(2) D1 = donepezil 1 mg/kg (i.p.).
(3) D3 = donepezil 3 mg/kg (i.p.).
(4) T3 = tacrine 3 mg/kg (i.p.).
(5) T5 = tacrine 5 mg/kg (i.p.).

Each animal represented an individual experimental unit. A total of 40 rats were included in the study. No animals or data points were excluded from the analysis.

To provide a statistical rationale for the group size, a sensitivity power analysis was performed in G*Power 3.1 for a one-way ANOVA design with five groups (α = 0.05, total *n* = 40), which indicated 80% power to detect a large effect size (Cohen’s f = 0.58) [26].

Donepezil doses (1 and 3 mg/kg) were selected based on previous rat behavioral studies using this dose range as behaviorally active in memory paradigms [27,28]. Tacrine doses (3 and 5 mg/kg) were chosen because both have previously been reported to produce significant behavioral effects in rat memory paradigms, with 3 mg/kg used as a lower active dose and 5 mg/kg included as a higher acute dose to explore potential dose-related effects associated with broader dual AChE/BuChE inhibition [29,30].

To reduce potential bias related to testing order or time of day, drug administration and behavioral testing were carried out in an alternating sequence across experimental groups. Thus, the first animal from each group was administered the corresponding treatment consecutively, in the following order: control, donepezil 1 mg/kg, donepezil 3 mg/kg, tacrine 3 mg/kg, and tacrine 5 mg/kg. The same sequence was then repeated for the second animal from each group, and continued accordingly until all animals had been treated and tested.

### 2.3. Substances

The following standardized preparations were used:

(1) Donepezil hydrochloride, 1 g vial, Sigma-Aldrich/Merck, St. Louis, MO, USA; product code: D6821.
(2) Tacrine hydrochloride hydrate—9-amino-1,2,3,4-tetrahydroacridine hydrochloride hydrate, 1 g vial, Sigma-Aldrich/Merck, St. Louis, MO, USA; product code: A79922.
(3) Sodium chloride 0.9% solution, 500 mL, B. Braun, Melsungen, Germany.

All substances were administered intraperitoneally. Drug solutions were freshly prepared to ensure a final injection volume of 0.1 mL/100 g body weight.

### 2.4. Y-Maze Apparatus

Exploratory behavior was assessed using a two-session Y-maze task. The apparatus consisted of a custom-made Y-shaped maze with three identical arms (47 cm in length, 15 cm in width, and 38 cm in height), constructed from wood and coated with adhesive foil to ensure a uniform surface texture.

The Y-maze was organized into three predefined arms: the start arm (S), the familiar arm (K), and the novel arm (U). During the acquisition session, access to the novel arm was prevented, whereas the start and familiar arms remained available. In the retention session, all three arms were accessible.

Testing was performed under controlled environmental conditions. The maze was evenly illuminated, and external noise was reduced as much as possible. To maintain stable spatial orientation, the apparatus was placed in a constant position relative to surrounding visual cues throughout the experiments. Its position was checked before testing to ensure that no unintended displacement or rotation had occurred.

Animal behavior was monitored by means of a video recording system. A camera placed near the lateral end of the start arm was connected to a computer in an adjacent room, allowing the experimenter to observe the session remotely. Recordings were saved and analyzed after the experiment, thereby reducing direct interference during testing and limiting observational bias.

### 2.5. Experimental Procedure

To ensure consistency, all animals were handled by the same experimenter. After placing each rat in the maze, the experimenter immediately left the testing room. Animals were randomly assigned to experimental groups. The experimenter responsible for drug administration was aware of group allocation during the injection phase. Video recordings were subsequently evaluated by an independent investigator who was unaware of the treatment assigned to each animal. Data analysis was conducted by a third independent investigator who was also blinded to group allocation.

For each rat, behavioral assessment in the Y-maze was performed over two sessions, each lasting 5 min. All treatments were administered as a single intraperitoneal injection before the acquisition session, and the first session was performed 50 min after drug administration. The second session, corresponding to the 24 h recall session, was performed 24 h after the initial Y-maze exposure and was not preceded by any further drug administration. The interval of 50 min between treatment administration and the first maze session was selected based on previous pharmacokinetic and pharmacodynamic studies showing brain penetration and central cholinergic effects of donepezil and tacrine within a comparable timeframe [31,32,33,34]. During post-administration monitoring, animals were observed for overt cholinergic signs, including salivation, tremor, diarrhea, or obvious behavioral distress. No such signs were recorded after tacrine administration.

In the acquisition session, animals were allowed to explore only two arms of the maze: the start arm (S) and the familiar arm (K). Access to the third arm, later defined as the novel arm (U), was prevented using a wooden barrier with the same texture and visual characteristics as the maze interior. To avoid side preference bias, the spatial assignment of the familiar and novel arms was counterbalanced across animals. Accordingly, the familiar arm was located on the right side for half of the animals in each group and on the left side for the remaining animals. During the 24-h retention session, the barrier was removed and all three arms were available for exploration, while the familiar/novel arm designation was kept identical to that used during acquisition.

Between trials, the maze floor was cleaned to remove excretions and residual odor cues. The apparatus was first wiped with a damp cloth and then dried with clean wipes before the next animal was tested.

### 2.6. Behavioral Analysis

Behavioral assessment was based on four predefined maze regions: the familiar arm (K), the novel arm (U), the start arm (S), and the central intersection area (I). During the acquisition session, time spent in the start arm (S) and accessible arm (K), as well as the number of entries into these arms, was recorded. During the 24 h session, the following parameters were measured: time spent in the familiar arm, novel arm, start arm, and intersection zone, as well as the number of entries into the familiar and novel arms and total arm entries. Using the recorded behavioral measures, the following derived parameters were determined: the U/K time ratio, calculated as the time spent in the novel arm relative to the time spent in the familiar arm; the U/K entry ratio, defined as the number of entries into the novel arm relative to entries into the familiar arm; and the average duration of each visit to the familiar and novel arms.

An entry was considered valid when both hind paws of the animal were completely positioned within the corresponding arm. The animal was considered to have exited an arm once its hind paws were no longer inside that arm.

U/K time and entry ratios were interpreted as relative indices of exploratory allocation between the novel and familiar arms during the recall session and were considered together with the corresponding absolute arm-specific measures. Mean time per entry was used as a complementary parameter reflecting visit duration, without direct inference regarding mnemonic processes.

In addition, within-animal differences between novel- and familiar-arm values (U − K) were calculated for both time spent and number of entries in order to further assess treatment-related shifts in arm preference pattern between arm types during the 24 h session.

### 2.7. Statistical Analysis

Statistical processing was carried out with IBM SPSS Statistics for Windows, version 29.0 (IBM Corp., Armonk, NY, USA). Results are presented as mean ± standard deviation (SD). A *p* value < 0.05 was considered statistically significant. Data normality was examined with the Shapiro–Wilk test, while variance homogeneity was assessed using Levene’s test.

The primary outcome was the U/K time ratio during the 24 h recall session, defined as the ratio between time spent in the novel arm and time spent in the familiar arm. This parameter was selected because it reflects the relative distribution of exploratory behavior between the two key arms of the Y-maze. Arm-specific time measures, entry-based variables, mean time per entry, total arm entries, and U − K differential variables were considered secondary or complementary outcomes.

Acquisition-phase parameters were analyzed using one-way ANOVA, including time spent in the start arm (S) and familiar arm (K), entries into these arms, and total arm entries.

For the 24 h recall session, differences among groups were initially assessed using one-way ANOVA for the predefined primary outcome and for secondary behavioral variables, including arm-specific times, arm entries, U/K entry ratio, total arm entries, and mean time per entry. When a significant overall effect was identified, Tukey’s post-hoc test was applied for multiple comparisons.

To further evaluate the distribution of exploration between familiar and novel arms, complementary two-way mixed repeated-measures analyses were performed for arm times and arm entries separately, using treatment as the between-subject factor and arm type (familiar vs. novel) as the within-subject factor. In addition, within-animal differential analyses (U − K) were calculated for both time spent and number of entries and were subsequently compared among groups using one-way ANOVA followed by Tukey’s post-hoc test.

Because several behavioral outcomes were interrelated, secondary analyses were interpreted as complementary indicators of exploratory behavior rather than as fully independent primary outcomes. To reduce the risk of overinterpreting multiple correlated comparisons, the statistical interpretation focused on the predefined primary outcome and on the convergence of related secondary measures, rather than on isolated significant findings. Effect sizes were reported for the main overall analyses, and 95% confidence intervals were provided for the principal post-hoc comparisons.

## 3. Results

### 3.1. Acquisition Session

Acquisition-session behavioral outcomes are shown in Table 1. No significant group effect was detected for the duration of exploration in either the start arm (S) or the accessible/familiar arm (K), as assessed by one-way ANOVA (*p* = 0.927 and *p* = 0.857, respectively). The number of entries into S and K was also not significantly different between groups (*p* = 0.963 and *p* = 0.298, respectively), and total arm entries remained comparable (*p* = 0.449). Overall, these findings indicate that the experimental groups were behaviorally similar during acquisition, with no evident imbalance in initial exploratory activity. However, while these results argue against major treatment-related differences in acquisition-session exploratory activity, they do not exclude more subtle effects on encoding or early consolidation processes.

### 3.2. Time-Dependent Measures During the 24 h Session

#### 3.2.1. U/K Time Ratio

**Figure 1:**
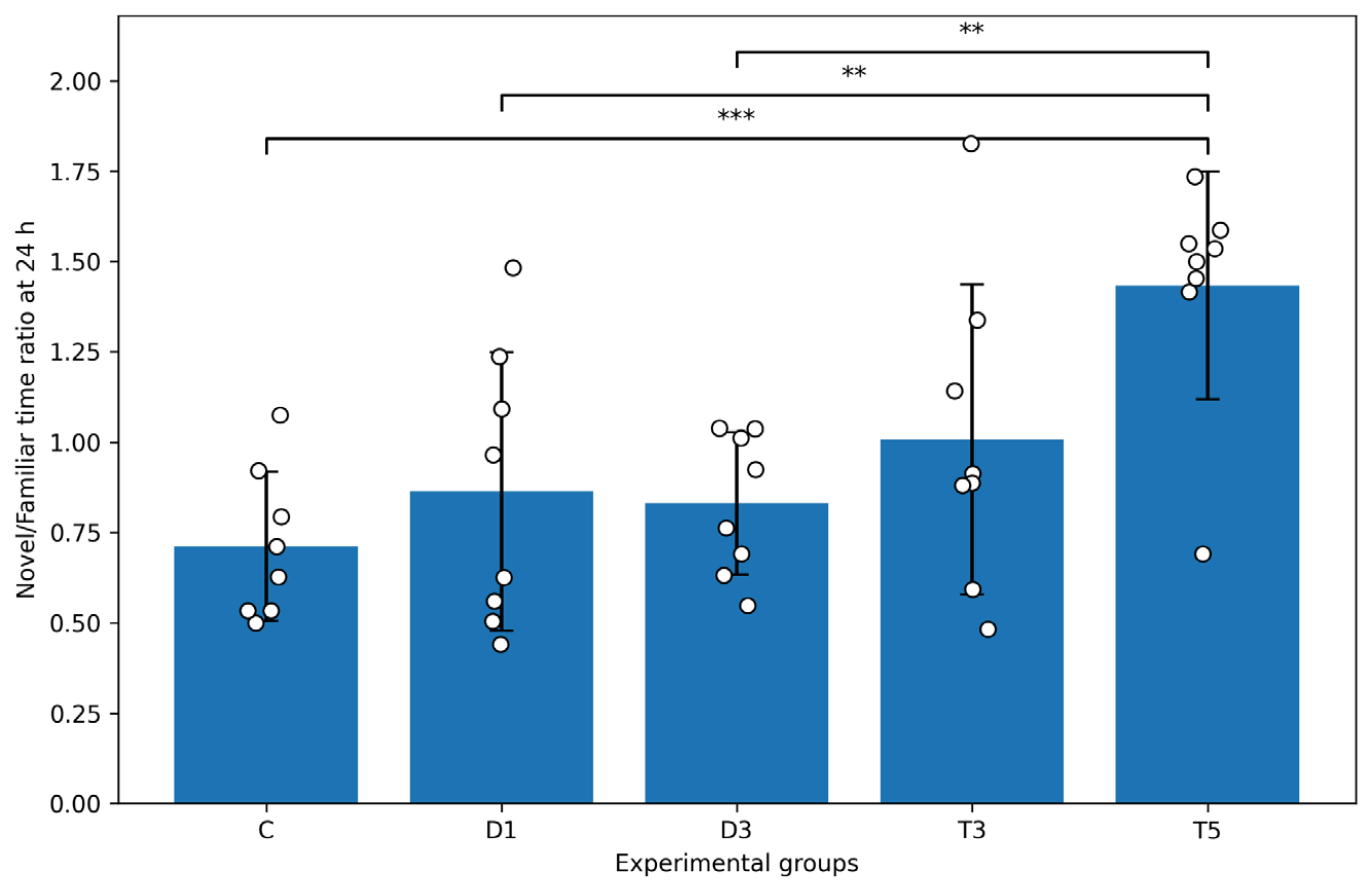
Mean individual U/K time ratio during the second trial (*n* = 8 per group). C = control group: 0.1 mL/100 g (i.p.), 0.9% saline, D1 = donepezil 1 mg/kg (i.p.), D3 = donepezil 3 mg/kg (i.p.), T3 = tacrine 3 mg/kg (i.p.), T5 = tacrine 5 mg/kg. Bars represent mean ± SD (mean of individual ratios); individual data points represent individual animals. The brackets indicate the comparisons and asterisks indicate the significance levels (** *p* < 0.01, *** *p* < 0.001). Statistical analysis: one-way ANOVA followed by Tukey’s post-hoc test.

The predefined primary outcome, the U/K time ratio during the recall session, is shown in Figure 1.

One-way ANOVA demonstrated a significant treatment effect on the U/K time ratio (F(4,35) = 6.11, *p* = 0.0008; η^2^ = 0.411; ω^2^ = 0.338). Post-hoc analysis revealed that the tacrine 5 mg/kg group showed a significantly higher U/K time ratio compared with the control group (mean difference = 0.721, 95% CI: 0.261 to 1.181, *p* = 0.0006), donepezil 1 mg/kg (mean difference = 0.569, 95% CI: 0.109 to 1.030, *p* = 0.0091), and donepezil 3 mg/kg (mean difference = 0.602, 95% CI: 0.142 to 1.062, *p* = 0.0052). No other significant differences were observed between groups.

These results suggest a relative shift in exploratory allocation, without implying enhanced novelty preference or a specific effect on memory performance.

#### 3.2.2. Time Spent in the Familiar and Novel Arms During the 24 h Session

**Figure 2:**
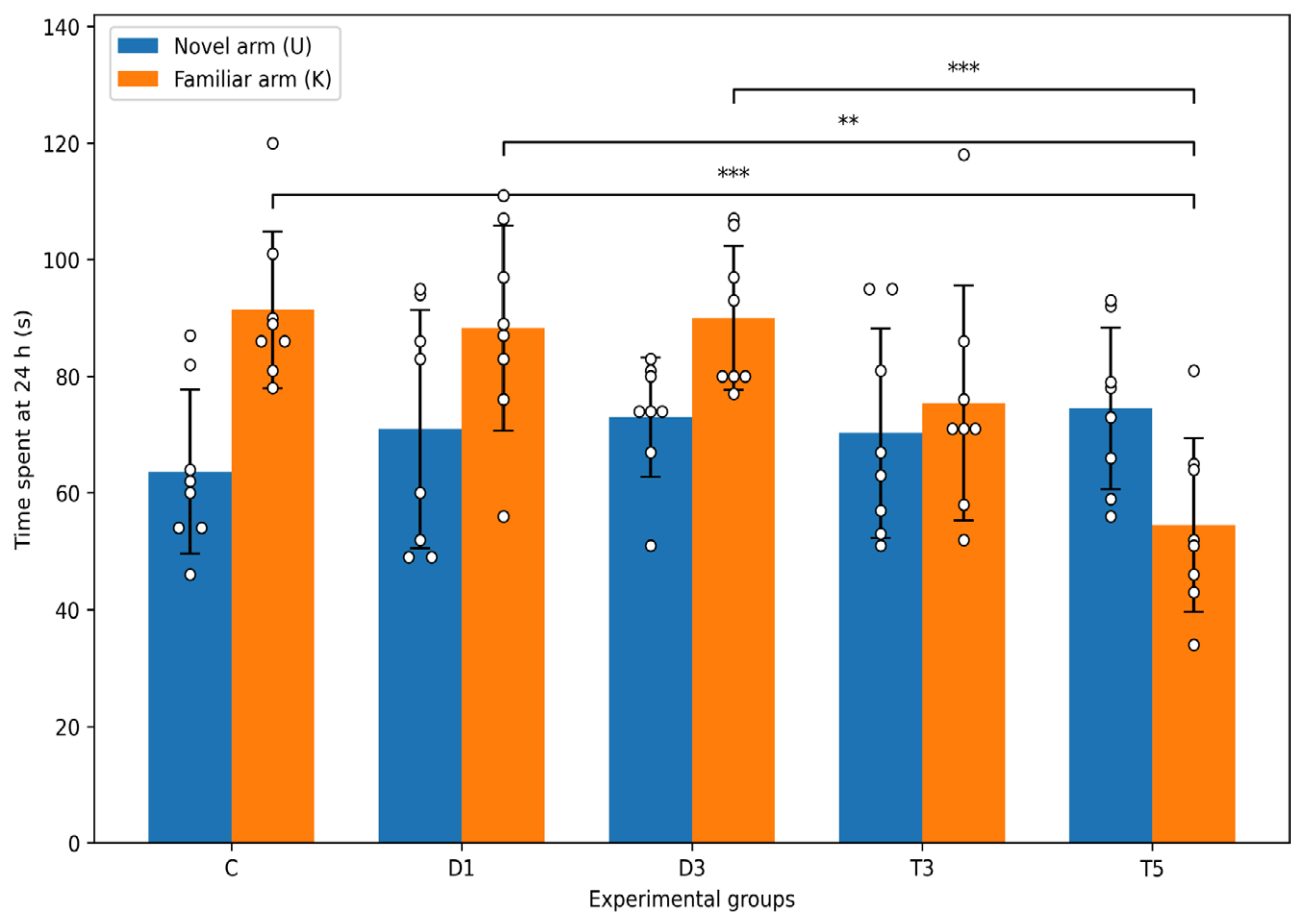
Time spent in Novel vs. Familiar Arms (Mean ± SD) (s) during the second trial (*n* = 8 per group)C = control group: 0.1 mL/100 g (i.p.), 0.9% saline, D1 = donepezil 1 mg/kg (i.p.), D3 = donepezil 3 mg/kg (i.p.), T3 = tacrine 3 mg/kg (i.p.), T5 = tacrine 5 mg/kg. Bars represent mean ± SD; individual data points represent individual animals. The brackets indicate the comparisons and asterisks indicate the significance levels (** *p* < 0.01, *** *p* < 0.001). Statistical analysis: one-way ANOVA followed by Tukey’s post-hoc test.

Mean time spent in the familiar (K) and novel (U) arms during the recall session (24 h) is shown in Table 2 and Figure 2.

In contrast to other groups, animals treated with tacrine 5 mg/kg spent less time in the familiar arm than in the novel arm.

A one-way ANOVA revealed a significant effect of treatment on time spent in the familiar arm (F(4,35) = 7.64, *p* < 0.001; η^2^ = 0.466; ω^2^ = 0.399). Post-hoc analysis (Tukey) showed that animals treated with tacrine 5 mg/kg spent significantly less time in the familiar arm compared with the control group (*p* = 0.0004), donepezil 1 mg/kg (*p* = 0.0014), and donepezil 3 mg/kg (*p* = 0.0007). No other significant between-group differences were observed.

For time spent in the novel arm, no significant treatment effect was detected (F(4,35) = 0.57, *p* = 0.689). Additional exploratory parameters, including time spent in the start arm, time spent in the intersection zone, combined time spent outside the familiar and novel arms, and total arm entries, are reported in Supplementary Table S1. These complementary data were included to contextualize overall maze activity and time allocation outside the two main analyzed arms.

Time spent in the familiar arm was significantly reduced in the tacrine 5 mg/kg group, whereas no significant differences were observed for time spent in the novel arm.

### 3.3. Entry-Dependent Measures During the 24 h Session

#### 3.3.1. Number of Entries into Familiar and Novel Arms

**Figure 3:**
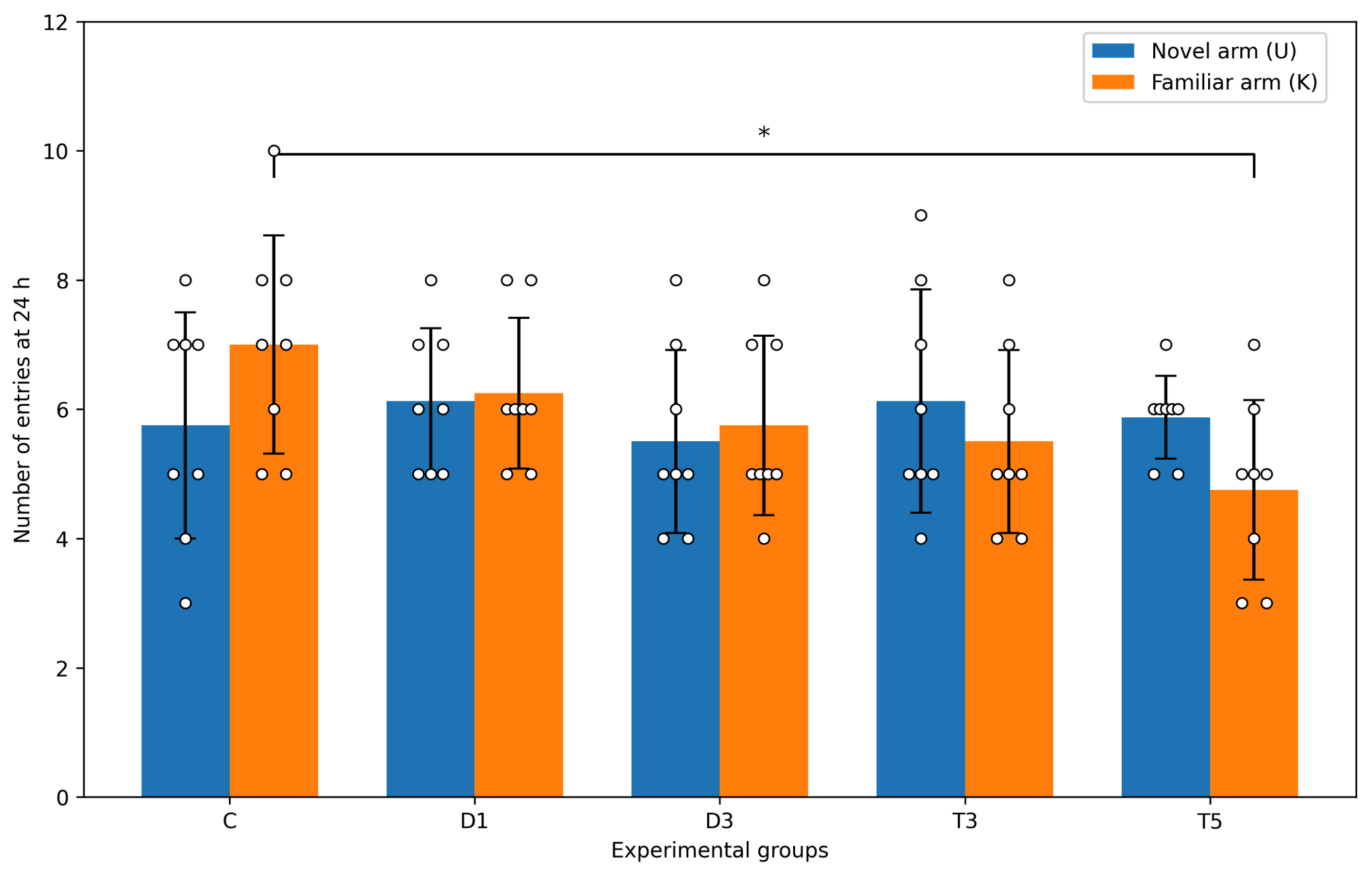
Number of entries into Novel vs. Familiar Arms (Mean ± SD) during the second trial (*n* = 8 per group). C = control group: 0.1 mL/100 g (i.p.), 0.9% saline, D1 = donepezil 1 mg/kg (i.p.), D3 = donepezil 3 mg/kg (i.p.), T3 = tacrine 3 mg/kg (i.p.), T5 = tacrine 5 mg/kg. Bars represent mean ± SD; individual data points represent individual animals. The brackets indicate the comparisons and asterisks indicate the significance levels (* *p* < 0.05). Statistical analysis: one-way ANOVA followed by Tukey’s post-hoc test.

The number of entries into the familiar and novel arms at 24 h is summarized in Table 3 and Figure 3.

Across experimental groups, the mean number of entries into the two arms showed limited variation. In the control group, animals entered the familiar arm more frequently than the novel arm. A similar pattern was observed in the donepezil 3 mg/kg group, whereas in the donepezil 1 mg/kg group the values were nearly equal, with a slight predominance of entries into the novel arm. In the tacrine 3 mg/kg and tacrine 5 mg/kg groups, the mean number of entries into the novel arm was higher than that into the familiar arm.

A significant treatment effect was detected for entries into the familiar arm (F(4,35) = 2.80, *p* = 0.040; η^2^ = 0.242; ω^2^ = 0.153). Post-hoc analysis showed that the tacrine 5 mg/kg group made significantly fewer entries into the familiar arm compared with controls (*p* = 0.025). No other between-group differences were observed.

No significant overall treatment effect was detected for entries into the novel arm (F(4,35) = 0.29, *p* = 0.883).

#### 3.3.2. U/K Entry Ratio

**Figure 4:**
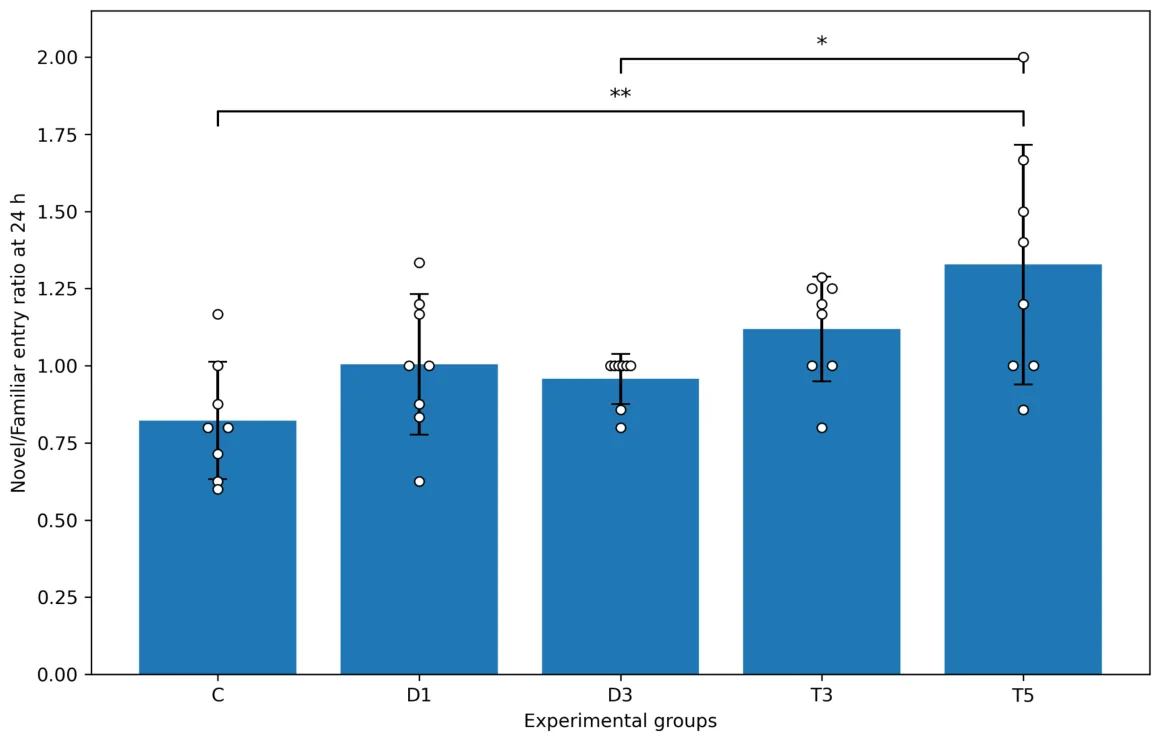
Mean U/K entry ratio during the second trial (*n* = 8 per group). C = control group: 0.1 mL/100 g (i.p.), 0.9% saline, D1 = donepezil 1 mg/kg (i.p.), D3 = donepezil 3 mg/kg (i.p.), T3 = tacrine 3 mg/kg (i.p.), T5 = tacrine 5 mg/kg. Bars represent mean ± SD; individual data points represent individual animals. The brackets indicate the comparisons and asterisks indicate the significance levels (* *p* < 0.05, ** *p* < 0.01). Statistical analysis: one-way ANOVA followed by Tukey’s post-hoc test.

The U/K entry ratio, analyzed as a secondary ratio-based outcome, differed significantly among groups (F(4,35) = 5.27, *p* = 0.002; η^2^ = 0.376; ω^2^ = 0.299) (Figure 4).

Post-hoc analysis indicated that the tacrine 5 mg/kg group exhibited a significantly higher ratio compared with the control group (mean difference = 0.505, 95% CI: 0.169 to 0.842, *p* = 0.001) and the donepezil 3 mg/kg group (mean difference = 0.371, 95% CI: 0.034 to 0.707, *p* = 0.025). No other significant differences were observed.

These findings show that the differences observed in the tacrine 5 mg/kg group were also reflected in ratio-based parameters.

### 3.4. Mean Time per Entry

**Figure 5:**
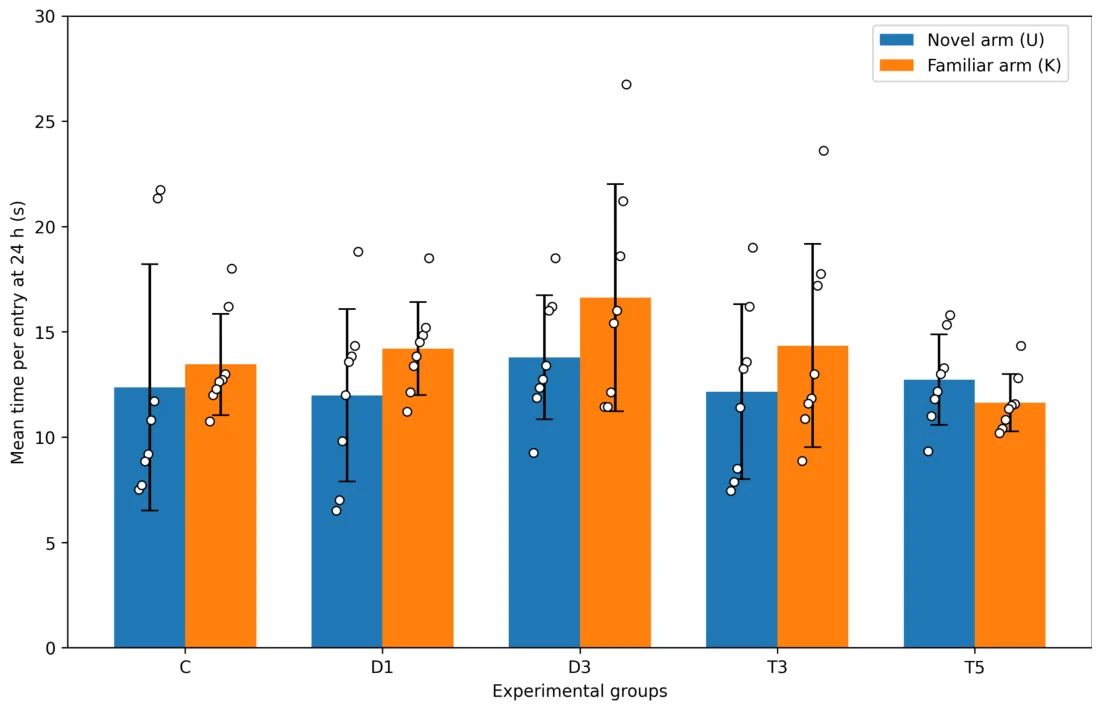
Mean time per entry into the familiar and novel arms (mean ± SD) (s) during the second trial (*n* = 8 per group)**.** C = control group: 0.1 mL/100 g (i.p.), 0.9% saline, D1 = donepezil 1 mg/kg (i.p.), D3 = donepezil 3 mg/kg (i.p.), T3 = tacrine 3 mg/kg (i.p.), T5 = tacrine 5 mg/kg. Bars represent mean ± SD; individual data points represent individual animals. Statistical analysis: one-way ANOVA followed by Tukey’s post-hoc test.

Mean time per entry into the familiar and novel arms is presented in Table 4 and Figure 5.

No significant treatment effects were observed for mean time per entry into the familiar arm (F(4,35) = 2.00, *p* = 0.117) or the novel arm (F(4,35) = 0.25, *p* = 0.906).

The absence of significant differences in this parameter suggests that the redistribution of exploration observed in the tacrine 5 mg/kg group was not accompanied by clear changes in visit duration per entry.

### 3.5. Complementary Treatment × Arm and Differential Analysis of Exploratory Allocation

To directly assess whether treatment modified the distribution of exploratory behavior between the familiar and novel arms during the 24 h session, a complementary two-way mixed repeated-measures analysis was performed, with treatment as the between-subject factor and arm type, novel versus familiar, as the within-subject factor. In addition, within-animal novel-minus-familiar arm differences (U − K) were calculated for both time spent and number of entries as convergent differential measures of exploratory profile.

For time allocation, the mixed repeated-measures analysis showed a significant treatment × arm interaction (F(4,35) = 4.06, *p* = 0.0096; partial η^2^ = 0.317), indicating that treatment modified the relative distribution of time between the novel and familiar arms. Consistently, the U − K time difference differed significantly across groups (one-way ANOVA, F(4,35) = 4.06, *p* = 0.0096; η^2^ = 0.311; ω^2^ = 0.227). Control animals showed negative U − K values, indicating greater time spent in the familiar arm, whereas tacrine-treated animals, particularly those receiving 5 mg/kg, showed a shift toward higher values. Post-hoc analysis identified a significant difference between the control and tacrine 5 mg/kg groups (mean difference = 47.75 s, 95% CI: 10.29 to 85.21, *p* = 0.0068).

A similar pattern was observed for arm entries. The mixed repeated-measures analysis showed a significant treatment × arm interaction for entries (F(4,35) = 4.74, *p* = 0.0037; partial η^2^ = 0.351), indicating that treatment also modified the relative distribution of entries between the two arms. Consistently, the U − K entry difference differed significantly among groups (one-way ANOVA, F(4,35) = 4.74, *p* = 0.0037; η^2^ = 0.351; ω^2^ = 0.272). Post-hoc testing showed significant differences between the control and tacrine 3 mg/kg groups (mean difference = 1.88 entries, 95% CI: 0.18 to 3.57, *p* = 0.0239), as well as between the control and tacrine 5 mg/kg groups (mean difference = 2.38 entries, 95% CI: 0.68 to 4.07, *p* = 0.0025).

Taken together, these complementary analyses support the interpretation that tacrine, particularly at the higher dose, altered arm preference pattern during the 24 h session by modifying the relative distribution of behavior between the familiar and novel arms.

## 4. Discussion

The present study provides a direct comparison of donepezil and tacrine under identical experimental conditions in healthy rats. Within this framework, tacrine, particularly at 5 mg/kg, produced the most pronounced changes in exploratory allocation during the second session of the Y-maze task. These observations should be interpreted within the limitations of a single behavioral paradigm.

Control animals did not exhibit a clear preference for the novel arm 24 h after acquisition, as time spent in the familiar arm exceeded that in the novel arm. Under baseline conditions, this pattern suggests a tendency toward persistence in the previously visited arm rather than pronounced novelty-driven exploration.

Donepezil (1 and 3 mg/kg) did not substantially modify this profile. No significant differences were observed compared with controls for time spent in either arm or for the U/K ratio. These findings suggest that selective acetylcholinesterase (AChE) inhibition in animals with an intact cholinergic system does not markedly influence exploratory allocation under these conditions. Moreover, cholinergic modulation likely follows a non-linear relationship, in which additional enhancement beyond physiological levels does not necessarily translate into measurable cognitive gains and may instead alter the balance between attention, arousal, and exploratory allocation.

In contrast, tacrine produced a dose-related behavioral modulation. While tacrine 3 mg/kg did not differ significantly from controls in the arm-wise time analysis, tacrine 5 mg/kg significantly reduced time spent in the familiar arm and increased the U/K time ratio. Importantly, this effect was not driven by a significant increase in absolute time spent in the novel arm, but rather by reduced familiar-arm exploration. Complementary exploratory parameters provided in Supplementary Table S1 further supported this cautious interpretation: total arm entries did not differ significantly among groups, whereas the tacrine 5 mg/kg group showed increased combined S + I time. Thus, the reduction in familiar-arm time reflected broader redistribution of time allocation across the maze rather than a selective increase in novel-arm exploration or a major change in overall exploratory activity. The complementary treatment × arm analysis and U − K differential analysis further supported this interpretation, showing that treatment modified the relative behavioral distribution between familiar and novel arms. Such findings may reflect altered exploratory preference between competing spatial options rather than a direct strengthening of mnemonic processes. However, because this pattern was not accompanied by increased absolute novel-arm exploration, non-mnemonic contributions cannot be excluded, including altered arousal, non-specific behavioral activation, changes in exploratory drive, or mild peripheral cholinergic discomfort.

This interpretation was supported by entry-based measures. Although entries into the novel arm did not differ significantly across groups, tacrine 5 mg/kg reduced entries into the familiar arm and significantly increased the U/K entry ratio. The concordance between time-based and entry-based indices indicates that the observed effects reflect a consistent shift in exploratory profile across complementary behavioral measures.

By contrast, mean time per entry did not differ among groups, suggesting that treatment effects were primarily associated with arm selection rather than with prolonged occupancy per visit. However, in the absence of independent locomotor or anxiety-related assessments, non-mnestic influences cannot be fully excluded.

Importantly, no statistically significant between-group differences were observed during the acquisition session, indicating that the treatment groups were broadly comparable during initial maze exposure. This reduces the likelihood that the behavioral differences observed during the 24 h session merely reflected marked baseline divergence already present at acquisition. However, because drug administration preceded acquisition, the present design does not allow a strict separation between effects on acquisition-related processing, encoding, consolidation, and later behavioral expression during the recall session.

The U/K ratios were interpreted together with the corresponding arm-specific measures. In the tacrine 5 mg/kg group, the higher U/K time and entry ratios were primarily associated with reduced exploration of the familiar arm, whereas absolute time spent in, and entries into, the novel arm did not differ significantly between groups. Thus, the ratio-based findings indicate a change in relative exploratory allocation rather than an absolute increase in novel-arm preference. The stronger effect observed with tacrine at 5 mg/kg may reflect its broader pharmacological profile [35,36,37,38,39,40]. In addition to inhibiting AChE and BuChE, tacrine has been reported to influence monoamine oxidase activity and slow outward K+ currents [38,39]. Because these pathways were not evaluated in the present study, the observed behavioral shift cannot be attributed exclusively to cholinergic modulation and may reflect a composite central pharmacodynamic effect rather than a specific modulation of mnemonic processes. These findings should be interpreted cautiously. Y-maze exploration may be influenced by non-mnestic factors such as locomotor activity, anxiety, novelty seeking, or general arousal. Although no significant differences were detected in mean time per entry, independent assessments of locomotor and anxiety-like behavior were not performed, limiting the specificity of interpretation. Future studies combining Y-maze testing with open-field or elevated plus maze paradigms would help disentangle mnemonic from affective and exploratory components [41,42,43].

Overall, under the identical experimental conditions used in the present study, tacrine exerted a more detectable influence on exploratory allocation in healthy rats than donepezil. This direct comparison provides a standardized behavioral reference for interpreting compound-specific effects in an intact cholinergic system. Rather than demonstrating unequivocal cognitive enhancement or a selective effect on a single memory stage, the data indicate a differential modulation of exploratory decision dynamics following cholinesterase inhibitor administration.

### Limitations

The present study has several limitations. First, behavioral interpretation was based on a single Y-maze paradigm, which does not allow unequivocal separation of mnemonic from non-mnemonic influences. Although the observed pattern is consistent with altered exploratory behavior during the 24 h session, the absence of complementary behavioral tests limits broader conclusions regarding the cognitive specificity of the observed effects.

Second, independent assessments of locomotor activity, anxiety-like behavior, or treatment tolerability were not performed. Therefore, potential contributions of arousal, exploratory drive, emotional reactivity, general activity levels or mild peripheral cholinergic discomfort, particularly at the higher tacrine dose cannot be fully excluded. In addition, although video scoring and data analysis were performed under blinded conditions, treatment administration itself was not blinded, as the experimenter administering the injections was aware of group allocation.

Third, the mechanistic interpretation remains limited by the absence of direct biochemical measurements. Neither AChE/BuChE activity nor other neurochemical correlates of cholinergic modulation were assessed; thus, the proposed interpretation based on differential cholinesterase selectivity should be considered plausible but was not directly demonstrated in the present study.

Finally, the study included only male rats. Male animals were selected in this exploratory design to reduce biological variability related to sex-dependent hormonal influences on behavioral performance; however, this choice limits the generalizability of the findings and future studies should include both sexes. In addition, individual housing and the routine institutional light cycle may have contributed to variability in exploratory behavior.

## 5. Conclusions

In this study, tacrine, particularly at 5 mg/kg, produced the clearest alteration in exploratory allocation in the two-trial Y-maze paradigm, characterized by reduced exploration of the familiar arm and increased U/K time and entry ratios. By contrast, donepezil produced only limited and non-significant behavioral changes under the present experimental conditions.

These findings provide a comparative characterization of the exploratory behavioral patterns associated with donepezil and tacrine in healthy rats. Because the changes observed with tacrine were not accompanied by a significant increase in absolute novel-arm exploration, they are best interpreted as alterations in exploratory allocation rather than as evidence of enhanced novelty preference or memory performance.

## Figures and Tables

**Table 1:** Behavioral measures recorded during the acquisition session. Values are expressed as mean ± SD; *n* = 8 animals per group. C = control group, 0.1 mL/100 g 0.9% saline, i.p.; D1 = donepezil 1 mg/kg, i.p.; D3 = donepezil 3 mg/kg, i.p.; T3 = tacrine 3 mg/kg, i.p.; T5 = tacrine 5 mg/kg, i.p.

Arm	C Mean ± SD	D1 Mean ± SD	D3 Mean ± SD	T3 Mean ± SD	T5 Mean ± SD
Time in S arm (s)	124.75 ± 29.01	121.88 ± 40.91	128.00 ± 38.17	122.63 ± 37.77	112.75 ± 23.74
Time in K arm (s)	112.25 ± 14.44	105.50 ± 27.50	121.00 ± 33.59	108.25 ± 34.37	117.12 ± 40.09
Entries into S arm	7.25 ± 1.83	6.25 ± 2.96	7.13 ± 3.80	6.75 ± 3.11	6.75 ± 2.38
Entries into K arm	8.25 ± 2.12	7.12 ± 2.59	6.00 ± 1.93	8.12 ± 3.04	8.00 ± 2.00

**Table 2:** Time spent in the Novel Arm (U) and Familiar Arm (K) during the recall session. Values are expressed as mean ± SD; *n* = 8 animals per group. C = control group, 0.1 mL/100 g 0.9% saline, i.p.; D1 = donepezil 1 mg/kg, i.p.; D3 = donepezil 3 mg/kg, i.p.; T3 = tacrine 3 mg/kg, i.p.; T5 = tacrine 5 mg/kg, i.p.

Arm	C Mean ± SD (s)	D1 Mean ± SD (s)	D3 Mean ± SD (s)	T3 Mean ± SD (s)	T5 Mean ± SD (s)
Novel arm (U)	63.62 ± 14.12	71.00 ± 20.44	73.00 ± 10.25	70.25 ± 17.92	74.50 ± 13.83
Familiar arm (K)	91.38 ± 15.14	88.25 ± 17.59	90.00 ± 12.38	75.38 ± 20.13	54.50 ± 14.86

**Table 3:** Number of entries in the Novel Arm (U) and Familiar Arm (K) during the recall session. Values are expressed as mean ± SD; *n* = 8 animals per group. C = control group, 0.1 mL/100 g 0.9% saline, i.p.; D1 = donepezil 1 mg/kg, i.p.; D3 = donepezil 3 mg/kg, i.p.; T3 = tacrine 3 mg/kg, i.p.; T5 = tacrine 5 mg/kg, i.p.

Arm	C Mean ± SD (Entries)	D1 Mean ± SD (Entries)	D3 Mean ± SD (Entries)	T3 Mean ± SD (Entries)	T5 Mean ± SD (Entries)
Novel arm (U)	5.75 ± 1.75	6.13 ± 1.13	5.50 ± 1.41	6.13 ± 1.73	5.88 ± 0.64
Familiar arm (K)	7.00 ± 1.69	6.25 ± 1.16	5.75 ± 1.39	5.50 ± 1.41	4.75 ± 1.39

**Table 4:** Mean time per entry in the Novel Arm (U) and Familiar Arm (K) during the recall session. Values are expressed as mean ± SD; *n* = 8 animals per group. C = control group, 0.1 mL/100 g 0.9% saline, i.p.; D1 = donepezil 1 mg/kg, i.p.; D3 = donepezil 3 mg/kg, i.p.; T3 = tacrine 3 mg/kg, i.p.; T5 = tacrine 5 mg/kg, i.p.

Arm	C Mean ± SD (s)	D1 Mean ± SD (s)	D3 Mean ± SD (s)	T3 Mean ± SD (s)	T5 Mean ± SD (s)
Novel arm (U)	12.36 ± 5.84	11.98 ± 4.10	13.79 ± 2.94	12.16 ± 4.16	12.71 ± 2.15
Familiar arm (K)	13.45 ± 2.40	14.20 ± 2.21	16.62 ± 5.39	14.34 ± 4.83	11.62 ± 1.36

## Data Availability

The data are available on request from the corresponding author.

## Supplementary Materials

The following supporting information can be downloaded at: https://www.mdpi.com/article/10.3390/biom16081102/s1, Table S1. Complementary exploratory parameters recorded during the 24 h session. Values are expressed as mean ± SD; *n* = 8 animals per group. C = control group, 0.1 mL/100 g 0.9% saline, i.p.; D1 = donepezil 1 mg/kg, i.p.; D3 = donepezil 3 mg/kg, i.p.; T3 = tacrine 3 mg/kg, i.p.; T5 = tacrine 5 mg/kg, i.p.; S = start arm; I = intersection zone. Time outside familiar and novel arms = combined time spent in the start arm and intersection zone, calculated as S + I.

## Abbreviations

The following abbreviations are used in this manuscript:

AD	Alzheimer’s disease
AChE	acetylcholinesterase
BuChE	butyrylcholinesterase
i.p.	intraperitoneal
ARRIVE	Animal Research: Reporting of In Vivo Experiments
SD	standard deviation
ANOVA	analysis of variance
