# Supplementary material for: Differential Effects of Donepezil and Tacrine on Recall-Phase Exploratory Behavior in Healthy Male Wistar Rats: A Two-Trial Y-Maze Study
Source: Biomolecules. 2026 Jul 28;16(8):1102. doi: 10.3390/biom16081102 (PMC13511269; doi:10.3390/biom16081102)
Supplement: Supplementary file 1 [file biomolecules-16-01102-s001.zip › biomolecules-4419283-supplementary.pdf]

biomolecules

MDPI

Supplementary Materials:

Table S1. Complementary exploratory parameters recorded during the 24 h session. Values are expressed as mean ± SD; *n* = 8 animals per group. C = control group, 0.1 mL/100 g 0.9% saline, i.p.; D1 = donepezil 1 mg/kg, i.p.; D3 = donepezil 3 mg/kg, i.p.; T3 = tacrine 3 mg/kg, i.p.; T5 = tacrine 5 mg/kg, i.p.; S = start arm; I = intersection zone; Time outside familiar and novel arms = combined time spent in the start arm and intersection zone, calculated as S + I.

Arm	C mean ± SD	D1 mean ± SD	D3 mean ± SD	T3 mean ± SD	T5 mean ± SD
Time in S arm (s)	78.25 ± 24.45	64.75 ± 30.80	65.50 ± 19.51	73.75 ± 20.36	69.25 ± 31.77
Time in I zone (s)	66.75 ± 23.71	76.00 ± 23.54	71.50 ± 21.93	80.63 ± 30.85	101.75 ± 28.53
Time outside familiar and novel arms (s)	145.00 ± 17.03	140.75 ± 17.38	137.00 ± 11.77	154.38 ± 19.77	171.00 ± 22.14
Total arm entries	19.50 ± 3.63	20.38 ± 2.97	17.88 ± 3.18	20.63 ± 3.07	19.13 ± 2.59
